# Serum proteomic profiling of precancerous gastric lesions and early gastric cancer reveals signatures associated with systemic inflammatory response and metaplastic differentiation

**DOI:** 10.3389/fmolb.2024.1252058

**Published:** 2024-03-22

**Authors:** Yueqing Gong, Yaxin Lou, Xiurui Han, Keyan Chen, Yang Zhao, Hejun Zhang, Jing Zhang, Ying Xiong, Weiwei Fu, Shigang Ding

**Affiliations:** ^1^ Department of Gastroenterology, Peking University Third Hospital, Beijing, China; ^2^ Beijing Key Laboratory for Helicobacter Pylori Infection and Upper Gastrointestinal Diseases (BZ0371), Beijing, China; ^3^ Medical and Health Analytical Center, Peking University, Beijing, China; ^4^ Department of Laboratory Medicine, Peking University Third Hospital, Beijing, China

**Keywords:** serum proteome, gastric cancer, *Helicobacter pylori*, intestinal metaplasia, atypical hyperplasia

## Abstract

The noninvasive detection technique using serum for large-scale screening is useful for the early diagnosis of gastric cancer (GC). Herein, we employed liquid chromatography mass spectrometry to determine the serum proteome signatures and related pathways in individuals with gastric precancerous (pre-GC) lesions and GC and explore the effect of *Helicobacter pylori* (*H. pylori*) infection. Differentially expressed proteins in GC and pre-GC compared with non-atrophic gastritis (NAG) group were identified. APOA4, a protein associated with metaplastic differentiation, and COMP, an extracellular matrix protein, were increased in the serum of patients with pre-GC lesions and GC. In addition, several inflammation-associated proteins, such as component C3, were decreased in the GC and pre-GC groups, which highlight a tendency for the inflammatory response to converge at the gastric lesion site during the GC cascade. Moreover, the abundance of proteins associated with oxidant detoxification was higher in the GC group compared with that in the NAG group, and these proteins were also increased in the serum of the *H. pylori*-positive GC group compared with that in the *H. pylori*-negative GC patients, reflecting the importance of oxidative stress pathways in *H. pylori* infection. Collectively, the findings of this study highlight pathways that play important roles in GC progression, and may provide potential diagnostic biomarkers for the detection of pre-GC lesions.

## 1 Introduction

Gastric cancer (GC) was the sixth most common cancer and fifth leading cause of cancer-related deaths worldwide in 2020 (Siegel et al., 2021). Due to its initial asymptomatic development and subsequent nonspecific symptoms, GC is often diagnosed at advanced stages with poor prognosis. Approximately two-thirds of patients present with incurable advanced or metastatic disease when diagnosed. Thus, early diagnosis of GC may increase the efficacy of treatment and improve the long-term survival of patients. To date, the most reliable diagnostic tool for GC is gastrointestinal endoscopy. However, this invasive technique is not suitable for large-scale screening. Moreover, serum-based gastrointestinal tumor markers, including the commonly used CEA, CA 19-9, and CA 72-4, are insufficient for the early diagnosis of GC due to their low sensitivity and specificity (Wobbes et al., 1992; Kochi et al., 2000; Ucar et al., 2008). Hence, an urgent need exists for the identification of clinically relevant noninvasive diagnostic markers.

Accordingly, research has been undertaken to characterize the serum proteome profile associated with GC and develop protein signatures to discriminate between healthy donors and patients with GC (Necula et al., 2019). However, no such proteome signature has been widely accepted nor applied in clinical practice, potentially due to the heterogeneity of GC.

According to the Correa model, intestinal-type GC is often initiated by *H. pylori* (*Helicobacter pylori*) infection and can develop via a cascade of chronic atrophic gastritis, intestinal metaplasia (IM), atypical hyperplasia (ATP), and ultimately carcinoma (Piazuelo and Correa, 2013; Moss, 2017). IM is defined as the replacement of normal gastric mucosa by intestinal epithelium, and is associated with an increased risk of ATP and cancer. ATP is closely synonymous to dysplasia and intraepithelial neoplasia, which includes low-grade intraepithelial neoplasia (LGIN) and high-grade intraepithelial neoplasia (HGIN). IM and ATP can be categorized as gastric precancerous (pre-GC) lesions. Although *H. pylori* eradication can reduce GC morbidity, it does not guarantee the prevention of GC development. This may be due to the pre-GC lesions that already exist. Thus, clarifying the molecular changes in pre-GC lesions may provide an opportunity for the early diagnosis of GC.

Recent transcriptome analyses have comprehensively explored gastric tumorigenesis, from inflammation through LGIN, and from HGIN to early GC (EGC) (Xu et al., 2014; Zhang et al., 2020). Single-cell transcriptome analyses have also identified premalignant gastric lesions and EGC (Zhang et al., 2019). However, proteomics remains a powerful approach for studying the molecular mechanisms of cancer, and the serum is a promising source of novel biomarkers for early detection. Moreover, a global proteomic analysis of serum samples from gastritis through the IM and ATP stages to EGC has not been previously reported. Furthermore, knowledge regarding the molecular changes that occur during GC development, including its development and mechanisms of progression, remains limited. Therefore, it is necessary to characterize the serum proteomes of patients with both pre-GC lesions and EGC.

In the current study, we performed a comprehensive analysis of the serum proteome using samples obtained from individuals with various gastric diseases, including pre-GC lesions and GC. Differentially abundant proteins and their enriched pathway characteristics between the IM/ATP and NAG groups, as well as the EGC and NAG groups were identified. Moreover, we compared the serum proteomes of *H. pylori*-positive and *H. pylori*-negative EGCs to identify *H. pylori*-related proteins and pathways. Finally, we explored what caused the changes of serum components in patients with GC and pre-GC lesions using the single-cell transcriptome data from human gastric tissue samples. Lastly, we evaluated the diagnostic value of the identified proteins which is coordinated with the expression levels in gastric tissue. We aim to identify serum signatures associated with GC and pre-GC lesions, which may also provide some insights into the intrinsic properties of GC carcinogenesis process.

## 2 Materials and methods

### 2.1 Participants

The patients were admitted to Peking University Third Hospital between August 2020 and April 2022. Thirty patients diagnosed with NAG, both IM and ATP, or EGC were enrolled. All patients were diagnosed according to the histological examination results, and *H. pylori* infection was identified using Warthin–Starry silver staining. Patients with other infections, autoimmune diseases, or other tumors were excluded from the study. The clinical characteristics of the patients are summarized in Table 1. Serum samples from routine clinical tests were collected as peripheral blood samples for analysis.

**TABLE 1:** Characteristics of enrolled patients with gastric lesions.

Characteristics	Gastritis (*n* = 7)	Premalignant lesions (*n* = 9)	Early gastric cancer (*n* = 14)
**Median age (years/range)**	51 (25–61)	73 (57–79)	64 (35–84)
**Sex**			
Male	4 (57.1%)	5 (55.6%)	11 (78.6%)
Female	3 (42.9%)	4 (44.4%)	3 (21.4%)
***Helicobacter pylori***			
Positive	1 (14.3%)	0 (0.0%)	4 (28.6%)
Negative	6 (85.7%)	9 (100.0%)	10 (71.4%)
**Histological diagnosis**	Chronic gastritis	Intestinal metaplasia with dysplasia	Early gastric cancer

### 2.2 Sample preparation

The sequential depletion of serum high-abundance proteins with dithiothreitol (DTT) and acetonitrile (ACN) was performed according to a published protocol (De et al., 2019). Briefly, 100 μL of serum was mixed with 11 μL of DTT and vortexed. The mixture was incubated at 37°C for 1 h followed by centrifugation. The supernatant was collected and diluted with 125 μL of 18.2 MΩ water prior to the addition of ACN. The mixture was sonicated and vortexed. ACN-precipitated proteins were pelleted by centrifugation. The supernatant was collected, evaporated to dryness, dissolved in UA buffer (8 M urea, 50 mM Tris, 75 mM NaCl, pH 8.5).

The proteins were digested with trypsin. Briefly, the proteins were added to a final concentration of 5 mM DTT and incubated at 56°C for 25 min. Proteins were alkylated in UA buffer supplemented with 14 mM iodoacetamide (IAA) for 30 min in the dark, followed by the addition of 5 mM DTT and incubation for 15 min at room temperature in the dark to quench unreacted IAA. The sample was loaded onto a Vivacon 500 device with a molecular weight cutoff (MWCO) of 10 kDa (Sartorius Stedim Biotech) and washed twice with 50 mM ammonium bicarbonate. Proteins were digested with trypsin in the same buffer at a 1:100 (enzyme:substrate) ratio at 37°C overnight. The digests were eluted and the filter was washed twice with 50 mM ammonium bicarbonate. The collected peptides were dried using a SpeedVac (Thermo Fisher Scientific, United States). All samples were pooled and fractionated using the Pierce™ High pH Reversed-Phase Peptide Fractionation Kit (ThermoFisher). Eleven fractions were collected, dried under vacuum, and prepared for data dependent acquisition (DDA) analysis.

### 2.3 NanoLC−MS/MS analyses

A Q-Exactive HF mass spectrometer coupled with an UltiMate 3,000 RSLCnano System (Thermo Fisher Scientific) was used for the DDA and data independent acquisition (DIA) experiments.

### 2.4 Mass spectrometry data analysis

DDA raw data were analyzed using Proteome Discoverer 2.2 (Thermo Fisher Scientific) against the Human UniProt FASTA database (20180528) to generate a library for DIA. The DIA data were analyzed using Skyline for protein identification and peak area calculations of proteins, according to the user guide (Egertson et al., 2015). Relative quantification of the proteins was performed using the MSstats tool.

### 2.5 Mass spectrometry data statistics

Statistical significance between groups was analyzed using MSstats (v 3.18.5) with default parameters. *p* < 0.05 and fold change>1.5 were considered statistically significant.

### 2.6 Immunoturbidimetric assay for C3 detection

The concentration of C3 in the patient serum was measured using an immunoturbidimetric assay kit (E032-1-1, NJJCBio, China). Briefly, the samples and standard solutions were incubated with C3 antibody at 37°C for 5 min. The increase in turbidity caused by the formation of insoluble immune complexes was measured to calculate the C3 concentration. Comparisons between groups were performed using t-tests.

### 2.7 ELISA for myeloperoxidase (MPO), cartilage oligomeric matrix protein (COMP), vitronectin (VTN), and glutaredoxin (GLRX) detection

The concentrations of MPO and COMP in the patient-derived serum were measured using specific ELISA kits based on the double antibody sandwich technique (ab119605 and ab213764, Abcam, USA). The concentrations of VTN and GLRX in the patient-derived serum were measured using ELISA kits (E10940 and E25374, Givei, China). Experiments were performed in accordance with the manufacturer’s instructions. The optical density at 450 nm was measured using a microplate reader. Comparisons between groups were performed using *t*-test, or one-way ANOVA, followed by Tukey’s HSD test.

### 2.8 Immunohistochemistry (IHC) for COMP and apolipoprotein A-IV (APOA4) detection in gastric tissue samples

The FFPE tissue samples include NAG (*n* = 10), IM (*n* = 16), and GC (*n* = 16). IHC was performed according to the instructions from the antibody manufacturer. Briefly, the FFPE slides of human gastric tissues were deparaffinized in xylene, and rehydrate by sequential incubation with 100%, 95%, 80%, and 60% ethanol. Then the slides were immersed with distilled water. The slides were transferred to sodium citrate buffer and heated for antigen retrieval. The slides were incubated with 3% H_2_O_2_ to quench endogenous peroxidase activity. The slides were blocked at room temperature for 1 h in 3% BSA PBS solution, and incubated with primary antibody solutions overnight at 4°C. APOA4 antibody (17996-1-AP, Proteintech, USA) and COMP antibody (28369-1-AP, Proteintech, USA) were diluted at ratio 1:200. Next, the slides were rinsed with PBST, incubated with secondary antibody, and then washed. The slides were incubated with DAB and washed. The slides were incubated with hematoxylin and washed. Next, the slides were immersed sequentially into 60%, 80%, 90%, and 100% ethanol bath, and then immersed in xylene. Finally, the slides were mounted with neutral balsam. The IHC image analyses were performed using ImageJ (v 1.5.1).

### 2.9 Pathway enrichment and protein interaction prediction

Conventional enrichment analysis and Gene Set Enrichment Analysis (GSEA) of Gene Ontology (GO) and KEGG pathways were performed using ClusterProfiler (Wu et al., 2021) (v 4.0).

The interactions of differentially expressed proteins (DEPs) were predicted using the STRING website tool (https://string-db.org) with the default parameter (Szklarczyk et al., 2019). The STRING networks were visualized by Cytoscape (v3.9.1).

### 2.10 Analysis of published microarray data

We obtained human RNA microarray data, GSE55696 (Xu et al., 2014) and GSE130823 (Zhang et al., 2020), derived from gastritis, LGIN and HGIN pre-GC lesions, and GC. Differential gene expression analysis was performed using the Limma package in the R language. Adjusted *p* < 0.05 was considered statistically significant.

### 2.11 Analysis of published single-cell transcriptome data

We obtained single-cell transcriptome data, GSE134520 (Zhang et al., 2019) and GSE150290 (Kim et al., 2022), derived from IM and GC. A standard single-cell RNA analysis procedure was performed using Seurat (Hao et al., 2021) (v4.1.1) to identify differentially expressed genes between the stages of gastric carcinogenesis. Statistical significance was set at adjusted *p* < 0.0001 (Wilcoxon rank-sum test).

### 2.12 Analysis using random forest model

We used random forest algorithm to construct a prediction model. This analysis was performed using “randomForest” R package, with default parameters (nTree = 500). The diagnostic value of this signature was demonstrated using receiver operating characteristics (ROC) analysis. The area under curve (AUC) values of ROC curves were calculated to estimate the predictive power of the models. The three-protein signature, APOA4-GCA-SERPINA4, was tested using the serum proteomic data, and then verified in the RNA array data, GSE55696 and GSE130823.

### 2.13 Data visualization

Figures and diagrams were produced using R with the ggplot2 and ComplexHeatmap packages. For the heatmaps and violin plots, missing-value imputation was performed using the K-Nearest Neighbors (KNN) algorithm (k = 5 using the NAguideR tool). The processed matrix was normalized using column sums. Enrichment network analysis and visualization were performed using Cytoscape.

## 3 Results

### 3.1 Serum proteome profiling analysis during a cascade of gastric lesions

**FIGURE 1:**
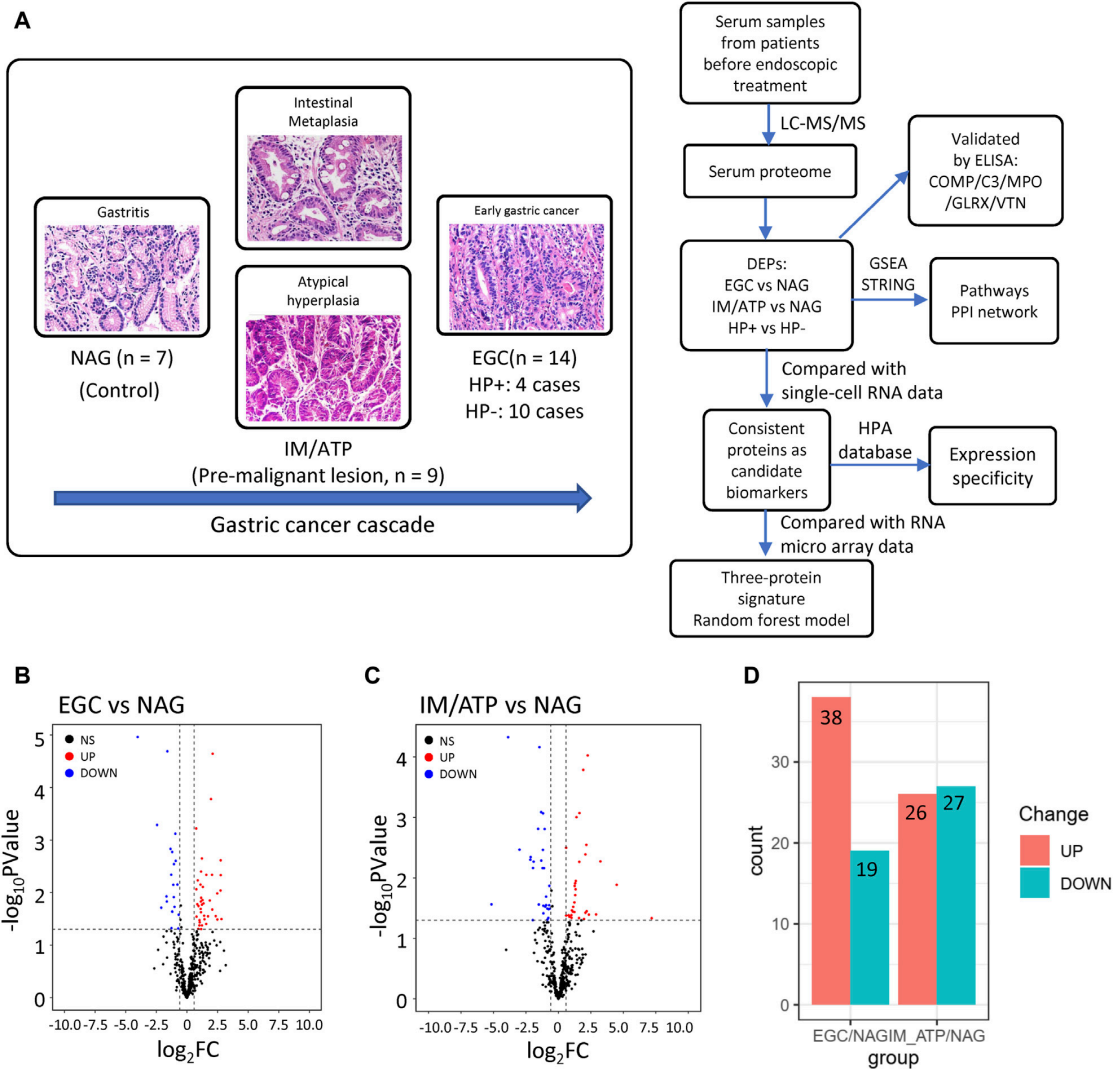
Overview of the serum proteome data from pre-GC lesions and EGC patients. **(A)** Protocol design for profiling serum proteins. **(B)** Volcano plot of EGC vs NAG. **(C)** Volcano plot of IM/ATP vs NAG. A *p* < 0.05 and fold change>1.5 were considered statistically significant. **(D)** Summary of upregulated and downregulated proteins in each comparison group.

To characterize serum proteome dynamics during GC development, serum samples were collected from patients with pathologically diagnosed gastric diseases at different stages: seven patients with NAG, nine patients with IM and ATP, and 14 patients with EGC (Figure 1A). The basic characteristics of the patients are summarized in Table 1. Relative quantification of protein expression levels was performed using the LFQ-Label Free quantification approach; 317 proteins were identified in the analyzed serum samples (Supplementary Table S1).

To further explore these data, DEPs were compared between groups. Fifty-seven proteins were found to be differentially expressed in EGC samples compared with their expression in patients with NAG (Figure 1B); 38 were upregulated and 19 were downregulated (Figure 1D and Supplementary Table S2). Fifty-three DEPs were identified in IM/ATP samples compared with their expression in patients with NAG (Figure 1C); 26 were upregulated and 27 were downregulated (Figure 1D and Supplementary Table S2).

### 3.2 Serum proteome profiling reveals several DEPs in the EGC group compared with their expression in the NAG group

Through differential protein expression analyses using MSstats, we identified many DEPs between the groups. We screened proteins with a 1.5-fold expression difference and statistical significance (*p* < 0.05) between different groups.

**FIGURE 2:**
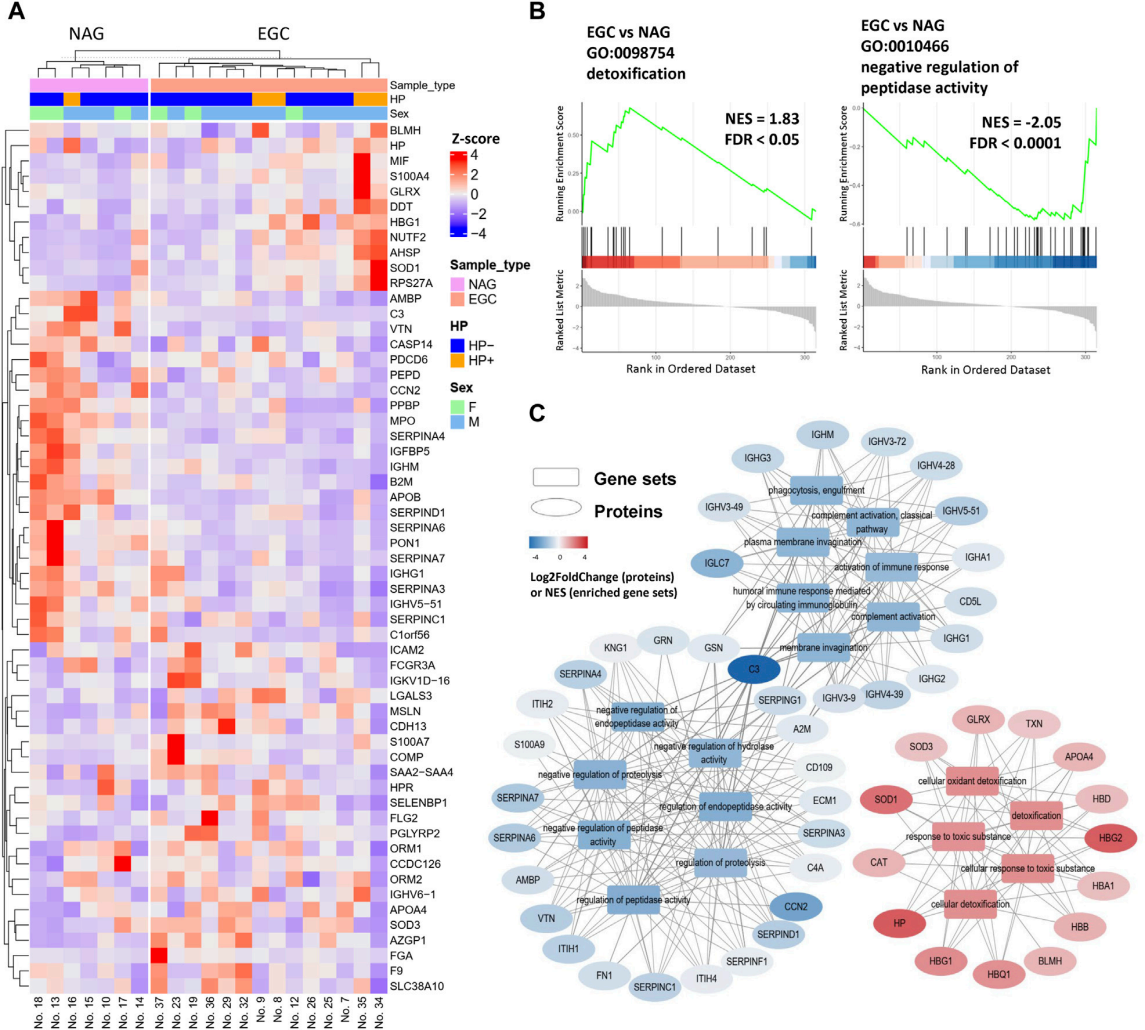
Differentially expressed proteins in the EGC group compared with their expression in the NAG group. **(A)** Heatmap of DEPs for the EGC vs NAG comparison group. **(B)** Enrichment plots of representative pathways. **(C)** The pathways can be classified into three categories: response to toxic substances, immune response, and regulation of peptidase activity.

First, we sought to determine whether serum proteome analysis could identify EGC-specific biomarkers. The EGC group had 57 proteins (38 upregulated and 19 downregulated) that significantly differed in abundance in comparison with the NAG group (Figure 2A). In addition to the previously reported potential biomarkers for GC detection, we found several molecules previously reported as being associated with GC. Caspase-14 (CASP14) and apolipoprotein B (APOB) expressions were decreased in the EGC group, while that of S100 calcium binding protein A7 (S100A7) was increased, compared with those in the NAG group (Figure 2A).

Furthermore, some proteins that reportedly participate in tumor development, but have not been evaluated in the context of GC, were detected. More specifically, nuclear transport factor 2 (NUTF2), GLRX, peptidoglycan recognition receptor 2 (PGLYRP2), and orosomucoid 2 (ORM2), were found to be elevated in GC for the first time in our study (Figure 2A). Hence, these proteins may be involved in GC development and serve as novel serum biomarkers for the early diagnosis and prognosis of GC.

To further identify changes in biological behavior during gastric tumorigenesis, GSEA (EGC group vs NAG group) was performed (Figures 2B, C). Protein-protein interaction (PPI) network analysis was also conducted using STRING database to understand the functional and physical association between the DEPs (Supplementary Figure S1). SERPINA7, SERPINA6, SERPIND1, SERPINA4, SERPINC1, and SERPINA3, were downregulated in the EGC group, all of which were involved in the negative regulation of the activity of serine proteases. In addition, the levels of component C3, MPO and paraoxonase 1 (PON1) were decreased in the EGC group (Figure 2A and Supplementary Figure S1). Using the STRING database, we found that PON1 was likely to interact physically with MPO—an inflammatory indicator (Rizo-Téllez et al., 2022). These results indicated altered inflammatory response in the EGC group.

The GSEA results also showed enrichment in cellular responses to toxic oxidant substances (Supplementary Figure S2 and Figure 2C). The normalized enrichment scores of the toxic substance response-associated gene sets were positive, which reflects enhanced resistance to toxic oxidative substances in the internal environment of patients with EGC (Figure 2C). We speculated that this originated from a protective response to the occurrence of gastric tumors or from the resistance of tumor cells to stress in the microenvironment. Traditional over-representation GO enrichment analysis was also performed to elucidate the functions of the DEPs (Supplementary Figure S3); the results were consistent with those of GSEA.

### 3.3 Analysis of DEPs in precancerous gastric lesions compared with their expression in the NAG group

**FIGURE 3:**
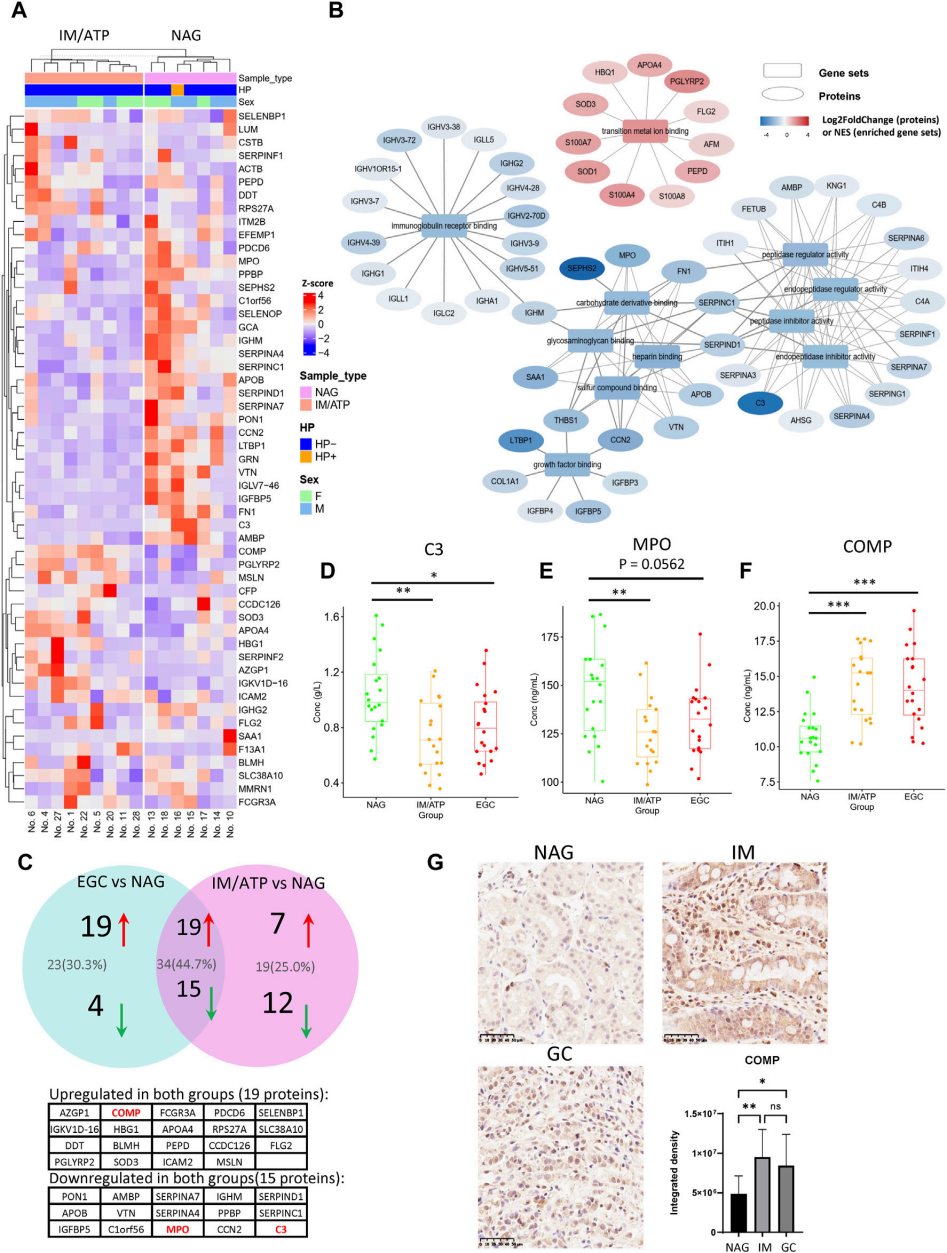
Differentially expressed proteins in the IM/ATP vs NAG group comparison. **(A)** Heatmap of differentially expressed proteins for the IM/ATP vs NAG comparison group. **(B)** Enriched GO-MF gene sets identified by GSEA. These pathways can be classified into five categories: transition metal ion binding, regulation of peptidase activity, metabolism of glycosaminoglycan, immune receptor binding, and growth factor binding. **(C)** DEPs of the IM/ATP vs NAG comparison overlaid with DEPs of the EGC vs NAG comparison. Thirty-four proteins were commonly upregulated or downregulated in the IM/ATP and EGC groups. **(D)** Serum concentration of component C3 (immunoturbidimetric assay). **(E)** Concentration of MPO in serum (ELISA assay). **(F)** Concentration of COMP in serum (ELISA assay). **(G)** COMP levels in the gastric tissue samples. These tissue samples include NAG (*n* = 10), IM (*n* = 16), and GC (*n* = 16). The barplot is the statistical result of the optical density analysis of IHC images. *p < 0.05; **p < 0.01.

IM and ATP are pre-GC lesions considered essential predisposing factors for GC development. Therefore, we further identified DEPs in the pre-GC group (individuals with IM and ATP) compared with their expression in the NAG group. Twenty-six upregulated and 27 downregulated DEPs were identified in the pre-GC group (Figure 3A). GSEA demonstrated that these DEPs were primarily enriched in transition metal ion binding, regulation of peptidase inhibitor activity, metabolism of glycosaminoglycan (such as heparin), immune receptor binding, and growth factor binding, with upregulated proteins involved in transition metal ion binding and downregulated proteins involved in immunoglobulin receptor binding, peptidase inhibitor activity, glycosaminoglycan metabolism, and growth factor binding (Supplementary Figure S4A and Figure 3B). The GSEA results for the representative gene sets are shown in Supplementary Figure S4B. Traditional GO analysis was performed to elucidate the functions of the DEPs (Supplementary Figure S5).

Notably, the DEPs and enrichment results of IM/ATP vs NAG partially overlapped with those of the EGC vs NAG comparison. In detail, 19 proteins were commonly upregulated and 15 were downregulated in the pre-GC and EGC groups (Figure 3C and Supplementary Table S3). In these two groups, the common downregulated proteins were enriched in immune responses and regulation of peptidase inhibitor activity. Certain downregulated DEPs in the IM/ATP group also exhibited physical interactions (Supplementary Figure S1), suggesting that the levels of these proteins are regulated by shared pathways. These findings indicate that the pre-GC lesions exhibited similar molecular changes as EGC.

Notably, our serum proteome data showed that the levels of C3 and MPO decreased in both the EGC and IM/ATP groups compared with those in the NAG group. Consistent results of C3 levels were verified by Enzyme-Linked Immunosorbnent Assay (ELISA) (Figure 3D). Similarly, the result also showed that the MPO concentration in the IM/ATP and EGC group was lower than that in the NAG group (Figure 3E). Other inflammation-related proteins, such as fibronectin 1 (FN1) (Wang et al., 2021) and grancalcin (GCA) (Li et al., 2021), also exhibited decreased expression in the IM/ATP group, while these two proteins were not significantly decreased in the EGC group.

Additionally, the level of COMP was increased in the EGC and IM/ATP groups compared with that in the NAG group, which was confirmed by ELISA (Figure 3F). We further detected the expression of COMP in gastric tissue by IHC, and the results showed that the level of COMP was also increased in tissue samples of EGC and IM group, compared with the samples of NAG group (Figure 3G). These results confirm that the serum COMP level is related to the early-stage progression of GC.

We also compared the serum proteomes of EGC and IM/ATP groups. Only a small number of DEPs were identified (Supplementary Table S2). Among these proteins, trefoil factor 3 (TFF3) was increased in the EGC group compared with that in the IM/ATP group, which is involved in multiple aspects of cancer progression (Yang et al., 2022) and has been reported to be increased in serum samples from patients with GC (Aikou et al., 2011) and more severe IM stages (Lee et al., 2022). That suggested that the increasing serum levels of TFF3 might indicate the progression from pre-GC stage to GC.

### 3.4 Differentially expressed proteins between *Helicobacter pylori*-positive and -negative EGCs

**FIGURE 4:**
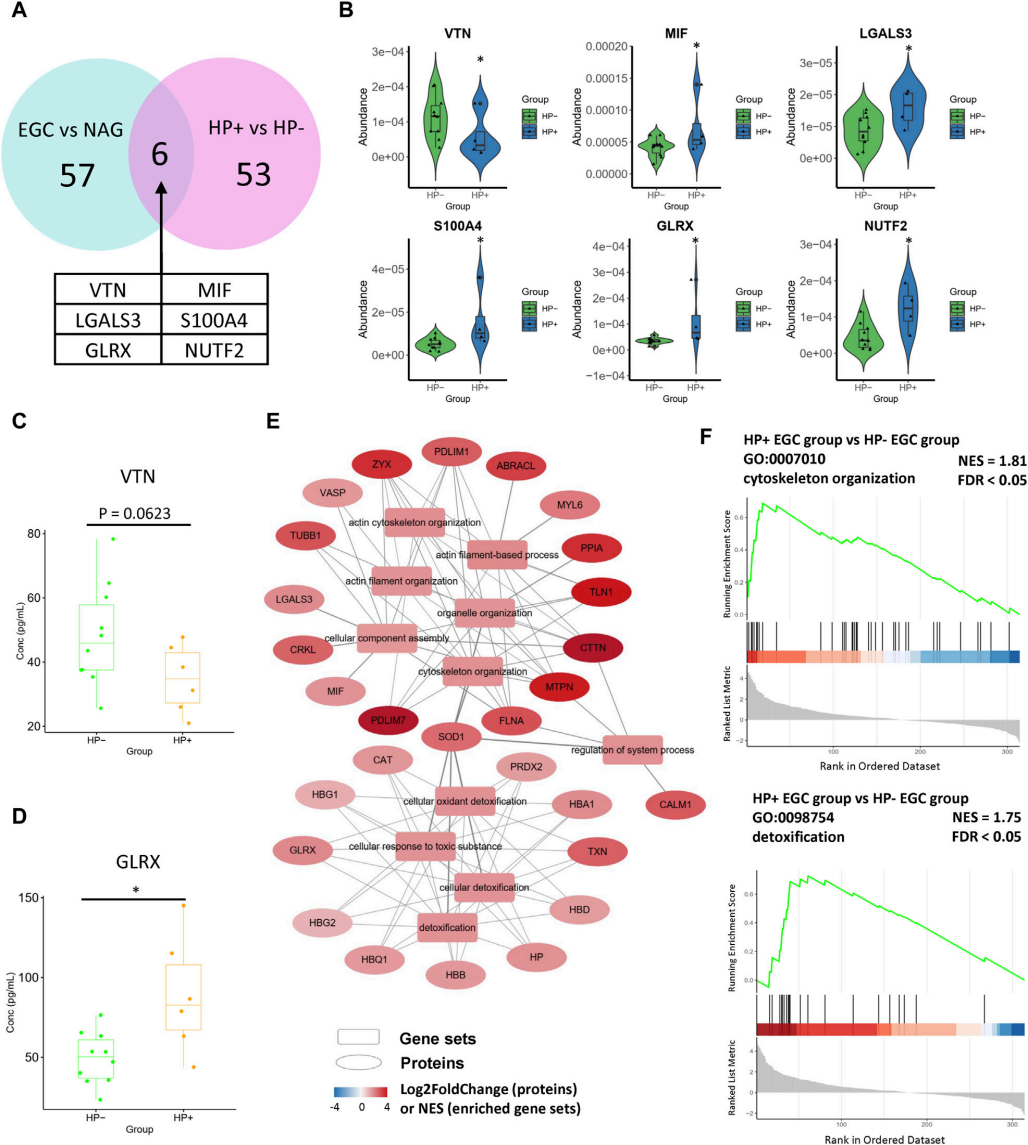
Differentially expressed proteins in the *Helicobacter pylori*-positive EGC group compared with their expression in the *Helicobacter pylori*-negative EGC group. **(A, B)** DEPs of the *Helicobacter pylori*-positive vs *Helicobacter pylori*-negative comparison overlaid with DEPs of the EGC vs NAG comparison. **(C)** Concentration of VTN in serum (ELISA assay). **(D)** Concentration of GLRX in serum (ELISA assay). **(E)** These pathways can be classified into two categories: cytoskeleton organization and response to toxic substance. **(F)** Enrichment plots of representative pathways.

Although *H. pylori* infection is a risk factor for GC initiation, to the best of our knowledge, the molecular mechanism of *H. pylori* infection in GC and its influence on the tumor microenvironment are largely unknown. Therefore, we also compared the serum proteomes of *H. pylori*-positive and *H. pylori*-negative EGC samples. A total of 26 proteins were upregulated in the *H. pylori*-positive GC group, while 27 were downregulated (Supplementary Figure S6). Notably, among DEPs of *H. pylori*-positive vs *H. pylori*-negative comparison group, six proteins were also the DEPs of the EGC vs NAG comparison group. Among the six proteins, migration inhibitory factor (MIF), Galectin 3 (LGALS3), S100A4, GLRX, and NUTF2 were upregulated, and VTN was downregulated in both comparison groups (Figures 4A, B). Consistently, ELISA results showed that the serum level of VTN was decreased in the *H. pylori*-positive GC group (Figure 4C), and GLRX level was increased in the *H. pylori*-positive GC group (Figure 4D).

Moreover, GSEA of the *H. pylori*-positive EGC group vs the *H. pylori*-negative EGC group (*H. pylori* comparison) revealed enrichment in cell motility-related pathways, including the actin filament-based process, actin cytoskeleton organization, and pathways related to detoxification (Supplementary Figure S7, Figures 4E, F), with upregulated DEPs. That is, the GSEA results of the *H. pylori* comparison also overlapped with those of the EGC vs NAG comparison. Traditional GO enrichment analysis also gave similar results. DEPs were particularly enriched in cellular oxidant detoxification, response to oxidative stress, nuclear protein transport, focal adhesion, immune response, and actin filament bundles in response to stress or infection (Supplementary Figure S8). Collectively, this consistency reflects the tumor-promoting effects of *H. pylori* infection, which may be associated with the oxidative stress in *H. pylori* infection.

### 3.5 Serum signatures associated with the gene expression levels in tissue

**FIGURE 5:**
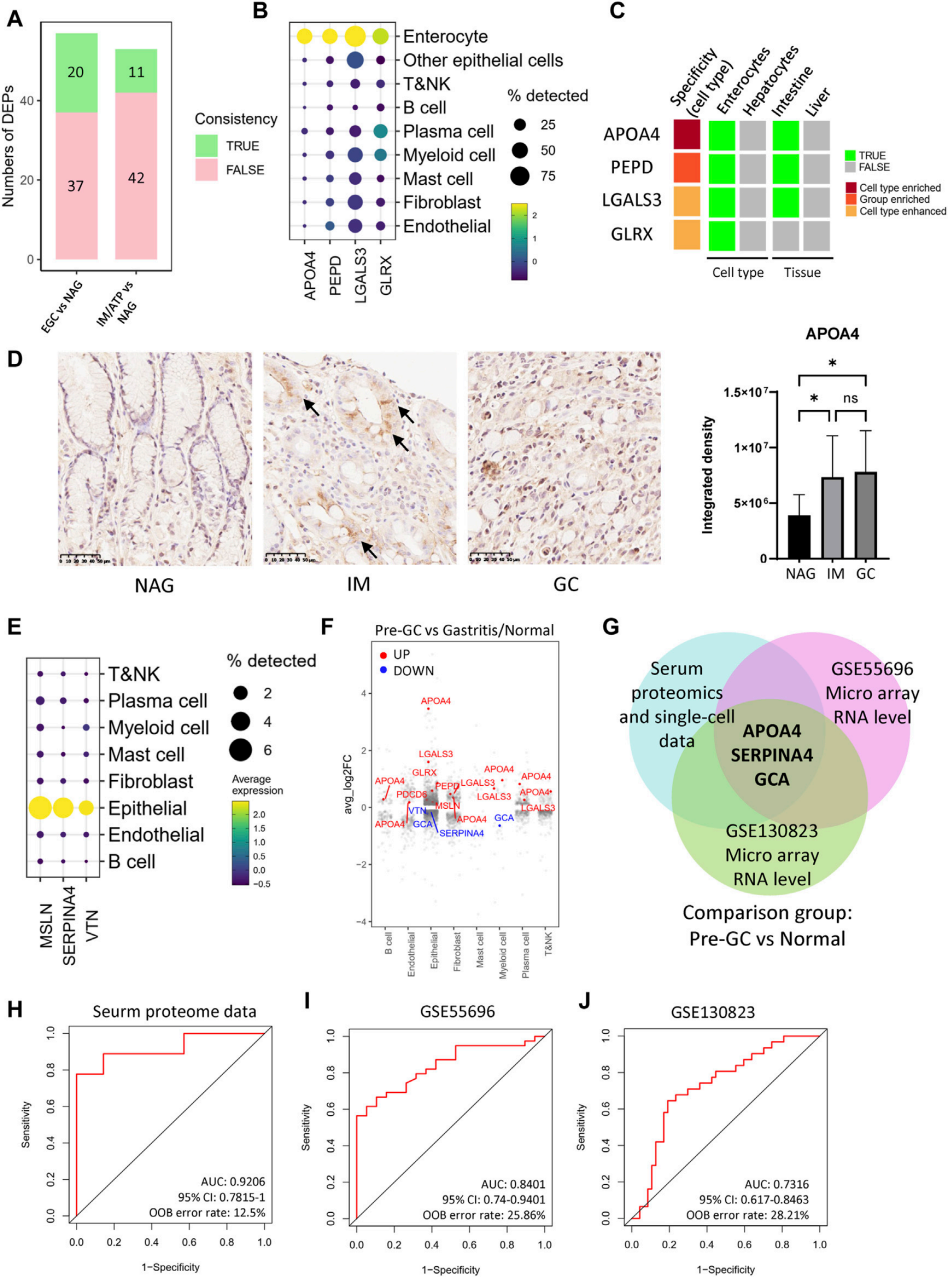
DEPs coincident with the tissue expression. **(A)** Numbers of DEPs coincident with the tissue expression. **(B)** DEPs which were mainly expressed by enterocytes in single-cell RNA data of gastric tissue samples. **(C)** DEPs with high expression specificity in enterocyte in Human Protein Atlas data. **(D)** APOA4 levels in the gastric tissue samples. The barplot is the statistical result of the optical density analysis of IHC images. These tissue samples include NAG (*n* = 10), IM (*n* = 16), and GC (*n* = 16). *p < 0.05; **p < 0.01. **(E)** DEPs which were mainly expressed by epithelial cells in single-cell RNA data. **(F)** Fold changes of DEPs across cell types in pre-GC vs gastritis/normal comparison group. **(G)** DEPs coincident with the RNA datasets, GSE55696 and GSE130823. **(H–J)** ROC curves of the random forest models using the signature, APOA4, SERPINA4, and GCA.

Generally, the changes of serum components in patients with GC and pre-GC lesions may be derived from the molecules released into the circulation by the gastric lesions. To further characterize the potential mechanism induced the changes of serum components in patients with GC and pre-GC, we attempted to select these proteins by comparing the serum proteomic data with the single-cell transcriptome data from gastric tissue samples. We screened out the differentially expressed genes (DEGs) by comparing the pre-GC/GC groups with the normal tissue/gastritis groups, using an integrated dataset from GSE134520 and GSE150290. The results showed that among 57 DEPs of EGC vs NAG comparison group, positive or negative log2FC of 20 DEPs were consistent with the corresponding DEGs; among 53 DEPs of IM/ATP vs NAG comparison group, positive or negative log2FC of 11 DEPs were consistent with corresponding DEGs (Figure 5A). These proteins were termed as “consistent proteins” (Supplementary Table S4). Notably, PDCD6, VTN, APOA4, PEPD, SERPINA4, and MSLN were consistent proteins for both IM/ATP vs NAG group and EGC vs NAG group, reflecting the consistent features of pre-GC and GC lesions (Supplementary Figure S9A). In addition, GCA, CSTB, DDT, ACTB, and MENT were the consistent proteins of IM/ATP vs NAG group, but were not the consistent proteins of EGC vs NAG group (Supplementary Figure S9A).

Next, we analyzed the single-cell data and used the Human Protein Atlas (HPA) database to understand the expression specificity in tissues and cell types. For the single-cell data, cell groups were identified using conserved marker genes (Supplementary Table S5, Supplementary Figures S9B, S9C, and 9D). The single-cell data showed that, among the consistent proteins, APOA4, PEPD, LGALS3, and GLRX were highly expressed in enterocytes, and APOA4 had the highest specificity (Figure 5B and Supplementary Figure S9E). In the process of intestinal metaplasia, the gastric mucosa suffers a conversion into an intestinal phenotype, and some epithelial cells go through metaplastic differentiation into enterocytes. Previous studies have shown that enterocytes existed in IM and GC lesions, but were hardly found in normal/gastritis tissues (Supplementary Figure S9F). The HPA data also showed that, for the organs and the cell types around the body, APOA4 expression is enriched in enterocytes, which is also one of the main cell types that expressed PEPD, LGALS3, and GLRX (Supplementary Table S6 and Figure 5C). These results suggest that the elevated serum levels of APOA4, PEPD, LGALS3 and GLRX may be caused by the enterocytes from metaplastic differentiation. We also detected the expression level of APOA4 in gastric tissue *in situ* by IHC. The results showed that APOA4 was significantly upregulated in GC and IM tissues (Figure 5D). Moreover, the APOA4 expression was enriched in the region of tubular structure which contained the metaplastic enterocytes and goblet cells (marked by arrow in Figure 5D). In addition, the single-cell data also showed that, in the pre-GC and GC lesions, MSLN, SERPINA4, and VTN were mainly expressed in the epithelial cells (Figure 5E), and their expression levels in the epithelial cells were consistent with the serum protein levels (Figure 5F and Supplementary Figure S9G). We also explored the features of DEPs which are not consistent with the single-cell RNA data. Among these proteins, only APOB and SELENBP1 are expressed in enterocytes, but are also expressed in hepatocytes (Supplementary Figure S9H). The results of enrichment analysis showed that these proteins were mainly associated with immune response, metabolic regulation, and responses to certain stresses (Supplementary Figure S9I).

To further demonstrating proteins which are useful as biomarkers of pre-GC lesions, we compared the consistent proteins of the pre-GC group from the serum proteomic data and single-cell data with another two RNA profiling datasets which contain pre-GC samples (Figure 5G). The results showed that APOA4, SERPINA4, and GCA were consistent in all datasets. SERPINA4 and GCA were both decreased in the IM/ATP group. The single-cell data showed that SERPINA4 was mainly expressed in epithelial cells (Figure 5E), and GCA was expressed in myeloid cells (Supplementary Figure S9J). We used a random forest model to test this three-protein signature in the serum proteomic data. The AUC value was 0.9206, suggesting this signature had a high diagnostic value for pre-GC lesions (Figure 5H). We also tested this signature in GSE55696 and GSE130823 micro array datasets, and the AUC values were 0.8401 and 0.7316, respectively (Figures 5I, J). Collectively, these results suggest that the proteins which are consistent with their expression levels in tissue may have high diagnostic value.

## 4 Discussion

In this study, we performed LC-MS/MS analysis and identified 57 and 53 DEPs in EGCs and pre-GCs, respectively. The identification of these proteins, including previously reported GC markers and those involved in GC or tumor development, demonstrated the effectiveness of our screening system. Moreover, some of the DEPs may serve as novel biomarker candidates for the diagnosis of GC. These proteins have not been previously evaluated in the context of GC. Reports on these proteins are summarized in Supplementary Table S7.

**FIGURE 6:**
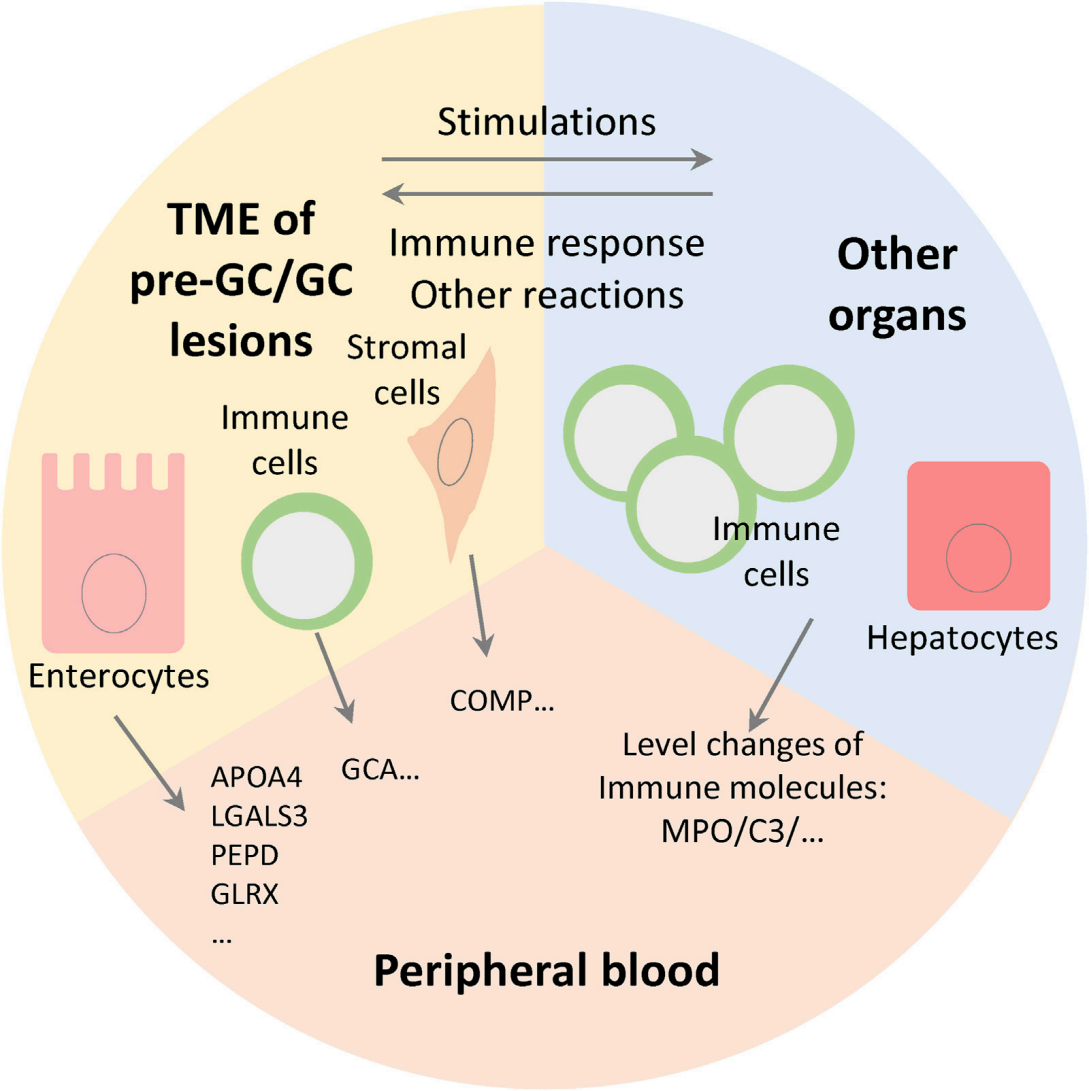
Level changes of serum protein components are determined by gene expression level in tissue and the systemic responses of immune system and other organs. The cells in tumor microenvironment (TME) and other organs together modulate the levels of blood protein components.

We investigated the changes of serum components in patients with GC and pre-GC lesions. Generally, pre-GC and GC lesions can release various molecules into the circulation; moreover, the progression of gastric lesions can cause some systemic responses, and in this process the abundance of serum components can be modulated by other organs, especially the liver and immune system (Figure 6). In the former case, the serum protein levels may be positively coordinated with the expression levels in gastric tissue. Notably, we found that a considerable number of DEPs were consistent with the tissue RNA data. Among these consistent proteins, the expression of APOA4 was highly enriched in enterocytes, and was upregulated in both EGC and IM/ATP groups, reflecting its potential diagnostic value. APOA4 is a component of high-density lipoprotein (HDL) and chylomicron and is related to the regulation of lipid metabolism. However, the relationship between APOA4 expression and tumor progression remains unknown. It was reported that APOA4 was decreased in the serum small extracellular vesicles of ovarian cancer patients (Lai et al., 2022), which implies that the relationships between APOA4 and tumor progression may be different among various cancer types.

The single-cell data also showed that some of the consistent proteins were enriched in other cell types. COMP is a matrix protein that stabilizes the extracellular matrix by interacting with collagen (Posey et al., 2018). In the tumor microenvironment (TME) of GC, COMP was mainly expressed in the fibroblasts. Notably, previous studies have demonstrated the tumor-promoting effects of COMP. TCGA, GTEx, and CCLE data showed that COMP was significantly overexpressed in 15 human cancers and was associated with tumor immune escape (Guo et al., 2023). Moreover, COMP could promote progression of hepatocellular carcinoma by activating the MEK/ERK and PI3K/AKT signaling pathways (Li et al., 2018). Emerging evidence showed that COMP also promoted breast cancer stem cell induction by activating Jagged1-Notch3 signaling (Papadakos et al., 2019), and accelerated breast cancer metastasis by modulating the metabolic process and metalloprotease-9 (MMP-9) secretion (Englund et al., 2016). Our data showed that COMP was upregulated in both EGC and IM/ATP groups, which suggested that COMP may be involved in the early stage of GC development.

Regarding proteins which were not consistent with the tissue RNA data, the changes of their serum levels may be induced by the systemic responses to cancer progression, which was confirmed by the enrichment analysis. These proteins are associated with immune responses, metabolic regulation, and other responses. Among these proteins, MPO, C3, and PON1 were commonly decreased in the pre-GC and EGC groups, and are associated with inflammation. The expressions of these proteins are closely related to an individual’s immune status and are, therefore, likely to fluctuate. MPO is an inflammatory indicator stored in the azurophilic granules of neutrophils. It acts as a microbicidal protein and modulates neutrophil function (Rizo-Téllez et al., 2022). C3 is a well-known factor in inflammation. Activated C3 is locally deposited in gastritis and GC lesions, and C3 deposition is negatively correlated with plasma C3 and C3a levels (Berstad et al., 1997; Yuan et al., 2020). C3 deposition is considered an immune signature for predicting GC prognosis. That is, high C3 deposition is an indicator of poor prognosis. The decreasing level of serum C3 reflected a tendency for the inflammatory response to converge in gastric lesions when progressing through the GC cascade. When passing through the cascade, blood C3 is likely activated and deposited in the tissue. PON1 is an antioxidant and anti-inflammatory glycoprotein that belongs to the paraoxonase family. It is mainly expressed in the liver and secreted into the bloodstream where it binds to HDL. Lower PON1 activity has been associated with several types of cancer, including GC (Meneses et al., 2019).

We also compared the serum proteomes of *H. pylori*-positive and negative EGC samples. *Helicobacter pylori* infection activates a number of pro-tumorigenic pathways (Zavros and Merchant, 2022). *Helicobacter pylori*-induced pathogenesis is largely associated with the chronic inflammatory response, which creates an immunosuppressive microenvironment, and causes atrophy followed by metaplasia. In addition, *H. pylori* virulence factors can promote the expression of CDX1 and CDX2, which suggest that *H. pylori* infection can promote the metaplastic differentiation process. Our results showed that the DEPs of *H. pylori*-positive and negative EGC samples partially overlapped with those of the EGC vs NAG group. These overlapped DEPs include MIF, LGALS3, S100A4, GLRX, NUTF2, and S100A4. Among these proteins, MIF and S100A4 are associated with immune response. Macrophage MIF controls the activity of proinflammatory cytokines and is associated with *H. pylori*-associated gastric carcinogenesis (Xia et al., 2004; Yoon et al., 2019). S100A4 is a member of the damage-associated molecular pattern family, and is overexpressed in many tumors and involved in tumor metastasis (Mishra et al., 2012). Patients with GC with high S100A4 expression showed lower 5-year overall and disease-specific survival (Treese et al., 2022). In addition, LGALS3 and GLRX are highly expressed in the enterocytes which are derived from metaplastic differentiation. LGALS3 is upregulated in gastric epithelial cells after *H. pylori* infection and promotes carcinogenesis (Subhash and Ho, 2016). It also counteracts the adhesion and exhibits chemoattraction to *H. pylori*-infected GC cells (Subhash et al., 2016). GLRX, which contributed to the antioxidant system, regulates the levels of glutathionylated proteins, and thus disrupt normal redox signaling (Ogata et al., 2021). GLRX silencing activated the p53 signaling pathway in tumor cell lines, and thus caused cell cycle arrest in G1 phase (Yang et al., 2018). The relationship between GLRX and GC remains unknown. VTN overexpression was associated with poor prognosis for GC (Gong et al., 2021), while our results showed that VTN was downregulated. The relationship between NUTF2 and tumor progression also remains unclear. Collectively, these results are consistent with the effect of *H. pylori* infection on the immune response and metaplastic differentiation process.

The DEPs of our serum proteomic study highlight the diagnostic value of enterocytes, which existed in pre-GC and GC lesions while hardly found in normal/gastritis tissues. Among the consistent proteins of EGC vs NAG group and IM/ATP vs NAG group, seven proteins were expressed in enterocytes. These results suggest that, as a specific cell group, enterocytes have high diagnostic value. In our three-protein signature of pre-GC lesions, APOA4 encodes an enterocyte-specific apolipoprotein, SERPINA4 encodes kallistatin, the inhibitor of tissue kallikrein, and GCA encodes grancalcin, a calcium-binding protein. As mentioned above, APOA4 represents the metaplastic differentiation process. Kallistatin, a tumor suppressor through multiple pathways (Chao et al., 2017), enhanced the expression of the tumorigenic suppressors, miR-34a and P53, in breast cancer cells (Li et al., 2016). It also suppressed the progression of colorectal cancer through binding to LRP6 (Shahbazi et al., 2023), and suppressed lymphatic metastasis by downregulating VEGF-C expression and secretion in GC (Ma et al., 2018). The plasma level of kallistatin was decreased in the GC group and negatively associated with the phase of lymph node metastasis. Our proteomic results suggest that the downregulated of kallistatin occurs in the pre-GC stage. The relationship between grancalcin and tumors remains unclear. Grancalcin may play a role in the adhesion of neutrophils to fibronectin, and thus modulate the immune response (Xu et al., 2006).

Collectively, our study reveals several serum DEPs and pathways that involved in both pre-GC and GC progression. Moreover, the serum characteristics of *H. pylori*-positive GC were demonstrated. These findings will inform the design of effective diagnostics. The proteins which are consistent with their expression levels in tissue may have high diagnostic value, because the serum levels of these proteins indicate the intrinsic properties of gastric lesions. A signature which contained three consistent proteins exhibited a high diagnostic value for pre-GC lesions. However, certain limitations were noted in this study, including the small sample size. Hence, to verify the specificity of potential markers, we performed additional verification using traditional immunoassays, such as ELISA and IHC. In addition to GC-related molecules, certain DEPs also have unknown functions in the GC cascade. Therefore, analysis using a larger sample size and exploration of the molecular functions are required to further confirm whether these candidates contribute to the diagnosis of EGC or pre-GC.

## Data Availability

The datasets presented in this study can be found in online repositories. The names of the repository/repositories and accession number(s) can be found in the article/Supplementary Material.
