# Supplementary material for: Serum proteomic profiling of precancerous gastric lesions and early gastric cancer reveals signatures associated with systemic inflammatory response and metaplastic differentiation
Source: Front Mol Biosci. 2024 Mar 22;11:1252058. doi: 10.3389/fmolb.2024.1252058 (PMC10995311; doi:10.3389/fmolb.2024.1252058)
Supplement: Supplementary file 3 [file Table7.DOCX]

Overview of the detected proteins and their relationships with gastric cancer in the previous studies

| Proteins | Symbols | Relationship with gastric cancer | References |
| --- | --- | --- | --- |
| Haptoglobin | HP | Haptoglobin is a highly sialylated glycoprotein containing four N-glycosylation sites. The abundance of all potential glycopeptide biomarkers was increased in gastric cancer patients. | [1] |
| Macrophage migration inhibitory factor | MIF | Within *H. pylor*i-positive group, MIF level was significantly elevated in patients with cancer than in control or dysplasia groups. | [2] |
| Fibrinogen alpha chain | FGA | High FGA expression indicates poor prognosis of gastric cancer. | [3] |
| Serum amyloid A | SAA | The serum level of SAA was higher in gastric cancer patients than in healthy subjects and gastric ulcer patients. | [4] |
| Mesothelin | MSLN | MSLN is upregulated in GC and predicts poor prognosis. MSLN promotes proliferation, migration, and invasion of gastric cancer cells. MSLN polymorphism, rs3764247 and rs3764246, were associated with a reduced risk of gastric cancer | [5] |
| Extracellular superoxide dismutase [Cu-Zn] | SOD3 | The level of SOD3 is increased in the serum of the gastric cancer patients. | [6] |
| Galectin-3 | LGALS3 | Galectin-3 plays a crucial role in promoting tumor-driven immune suppression. For gastric cancer, dual blockade of Galectin-3 and CD47 collaboratively suppressed tumor growth, increased phagocytosis, repolarized macrophages, and boosted T cell immune responses. | [7] |
| Orosomucoid 1 | ORM1 | The level of ORM1 is increased in the serum of the gastric cancer patients. | [6] |
| Pro-platelet basic protein | PPBP | Plasm level of PPBP RNA is a potential biomarker for gastric cancer detection. | [8-10] |
| Protein S100-A4 | S100A4 | S100A4 is a member of the damage-associated molecular pattern (DAMP) family, and is overexpressed in many tumors and is involved in tumor metastasis. The scRNA analysis reveals that S100A4 is upregulated in gastric cancer cells. Gastric cancer patients with high S100A4 expression showed lower 5-year overall and disease-specific survival. | [11-13] |
| Superoxide dismutase [Cu-Zn] | SOD1 | Cu/ZnSOD activity was significantly lower in adenocarcinoma and HP tissues when compared to the healthy control. SOD1-7958A/- genotype increases the risk of gastric cancer in Chinese Han population. | [14, 15] |
| Low affinity immunoglobulin gamma Fc region receptor III-A | FCGR3A | FCGR3A may serve as an immuno-oncogenic molecule that correlates with tumor immune infiltration levels and affects drug sensitivity; thus, it can serve as a promising biomarker for cancer detection, prognosis, therapeutic design, and follow-up. FCGR3A can serve as a candidate biomarker for Epstein-Barr virus (EBV)-associated gastric carcinoma. | [16, 17] |

**Reference**

1. Jeong, S., et al., *Detection of Aberrant Glycosylation of Serum Haptoglobin for Gastric Cancer Diagnosis Using a Middle-Up-Down Glycoproteome Platform.* J Pers Med, 2021. **11**(6).

2. Yoon, K., et al., *Correlation between macrophage migration inhibitory factor and autophagy in Helicobacter pylori-associated gastric carcinogenesis.* PLoS One, 2019. **14**(2): p. e0211736.

3. Duan, S., et al., *Novel prognostic biomarkers of gastric cancer based on gene expression microarray: COL12A1, GSTA3, FGA and FGG.* Mol Med Rep, 2018. **18**(4): p. 3727-3736.

4. Chan, D.C., et al., *Evaluation of serum amyloid A as a biomarker for gastric cancer.* Ann Surg Oncol, 2007. **14**(1): p. 84-93.

5. Shen, K., et al., *Polymorphisms of an oncogenic gene, mesothelin, predict the risk and prognosis of gastric cancer in a Chinese Han population.* Arch Toxicol, 2022. **96**(7): p. 2097-2111.

6. Subbannayya, Y., et al., *Identification of differentially expressed serum proteins in gastric adenocarcinoma.* J Proteomics, 2015. **127**(Pt A): p. 80-8.

7. Fan, Y., et al., *Galectin-3 Cooperates with CD47 to Suppress Phagocytosis and T cell Immunity in Gastric Cancer Peritoneal Metastases.* Cancer Res, 2023.

8. Wu, F., C. Chen, and F. Peng, *Potential Association Between Asthma, Helicobacter pylori Infection, and Gastric Cancer.* Front Oncol, 2021. **11**: p. 630235.

9. Chen, L., et al., *Identification of Combinations of Plasma lncRNAs and mRNAs as Potential Biomarkers for Precursor Lesions and Early Gastric Cancer.* J Oncol, 2022. **2022**: p. 1458320.

10. Su, C., et al., *Identification of plasma RGS18 and PPBP mRNAs as potential biomarkers for gastric cancer using transcriptome arrays.* Oncol Lett, 2019. **17**(1): p. 247-255.

11. Bian, S., et al., *Integrative single-cell multiomics analyses dissect molecular signatures of intratumoral heterogeneities and differentiation states of human gastric cancer.* Natl Sci Rev, 2023. **10**(6): p. nwad094.

12. Treese, C., et al., *S100A4 Is a Strong Negative Prognostic Marker and Potential Therapeutic Target in Adenocarcinoma of the Stomach and Esophagus.* Cells, 2022. **11**(6).

13. Mishra, S.K., H.R. Siddique, and M. Saleem, *S100A4 calcium-binding protein is key player in tumor progression and metastasis: preclinical and clinical evidence.* Cancer Metastasis Rev, 2012. **31**(1-2): p. 163-72.

14. Monari, M., et al., *Implications of antioxidant enzymes in human gastric neoplasms.* Int J Mol Med, 2009. **24**(5): p. 693-700.

15. Yi, J.F., et al., *Mn-SOD and CuZn-SOD polymorphisms and interactions with risk factors in gastric cancer.* World J Gastroenterol, 2010. **16**(37): p. 4738-46.

16. Li, L., et al., *Integrative Pan-Cancer Analysis Confirmed that FCGR3A is a Candidate Biomarker Associated With Tumor Immunity.* Front Pharmacol, 2022. **13**: p. 900699.

17. Deng, S.Z., et al., *Exploration of the Tumor Immune Landscape and Identification of Two Novel Immunotherapy-Related Genes for Epstein-Barr virus-associated Gastric Carcinoma via Integrated Bioinformatics Analysis.* Front Surg, 2022. **9**: p. 898733.
