# Supplementary material for: Serum proteomic profiling of precancerous gastric lesions and early gastric cancer reveals signatures associated with systemic inflammatory response and metaplastic differentiation
Source: Front Mol Biosci. 2024 Mar 22;11:1252058. doi: 10.3389/fmolb.2024.1252058 (PMC10995311; doi:10.3389/fmolb.2024.1252058)
Supplement: Supplementary file 5 [file DataSheet1.PDF]

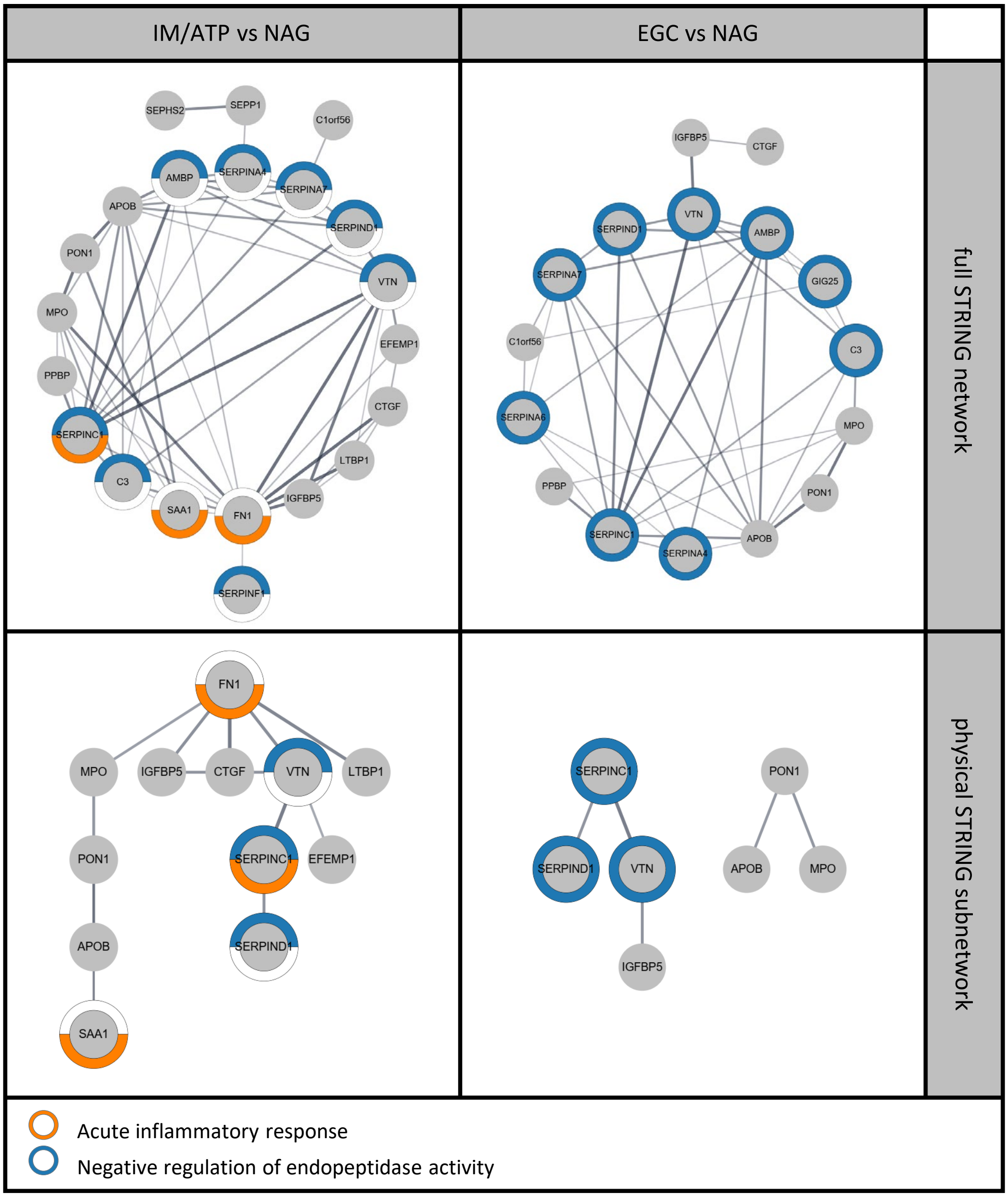

Figure S1. Physical interactions as well as functional associations of the downregulated DEPs. Querying STRING database, the functional associations and physical interactions of these proteins is exhibited by full STRING networks and physical subnetworks. The functions of the DEPs revealed by enrichment analysis is marked by orange and/or blue rings. Line thickness indicates the strength of data support (STRING score).

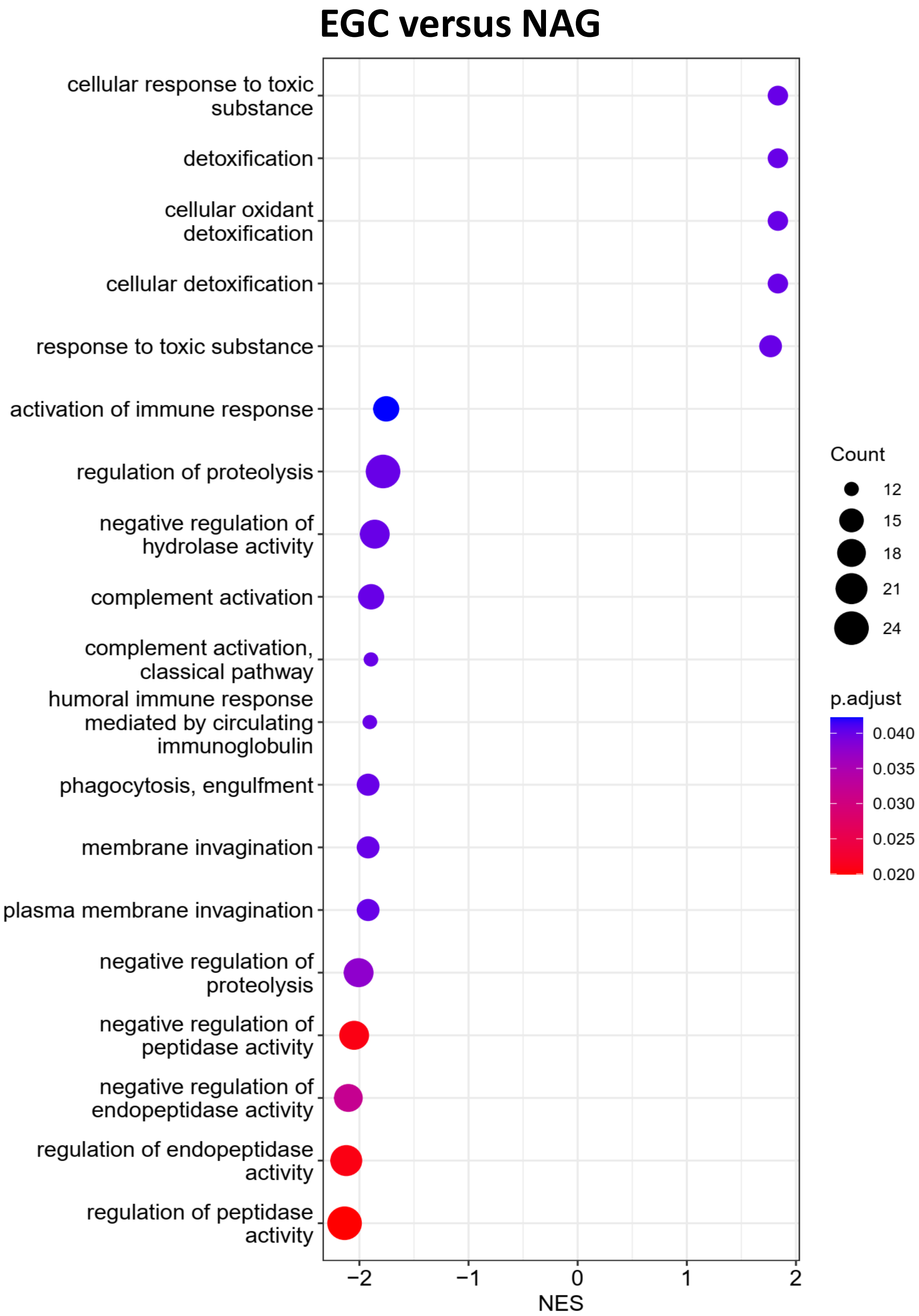

Figure S2. The enriched GO-BP gene sets by GSEA. The significantly enriched pathways (adjusted P value < 0.05) are listed.

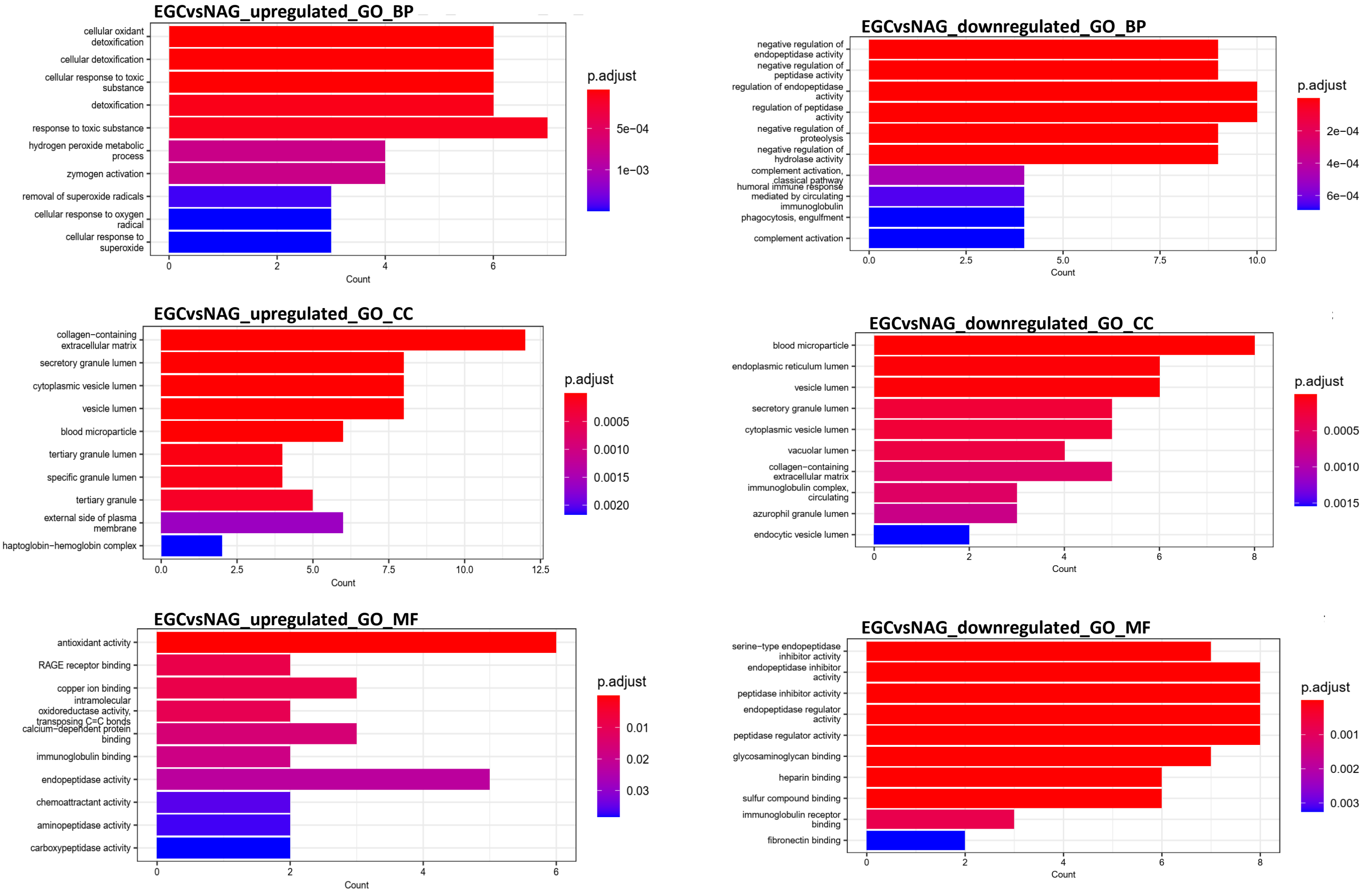

Figure S3. The traditional GO enrichment results of the EGC vs NAG group.

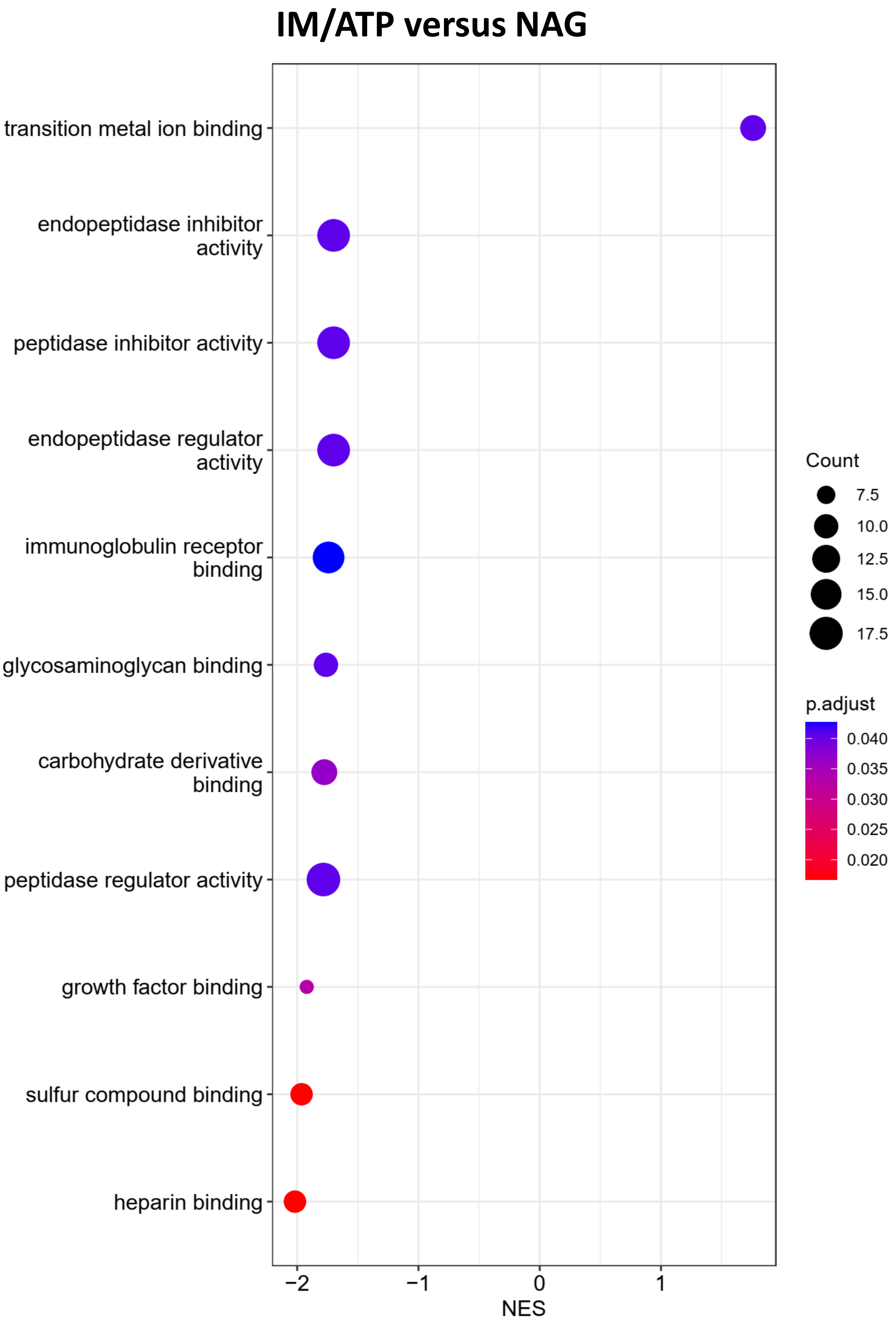

Figure S4. The enriched GO-MF gene sets by GSEA. The significantly enriched pathways (adjusted P value < 0.05) are listed.

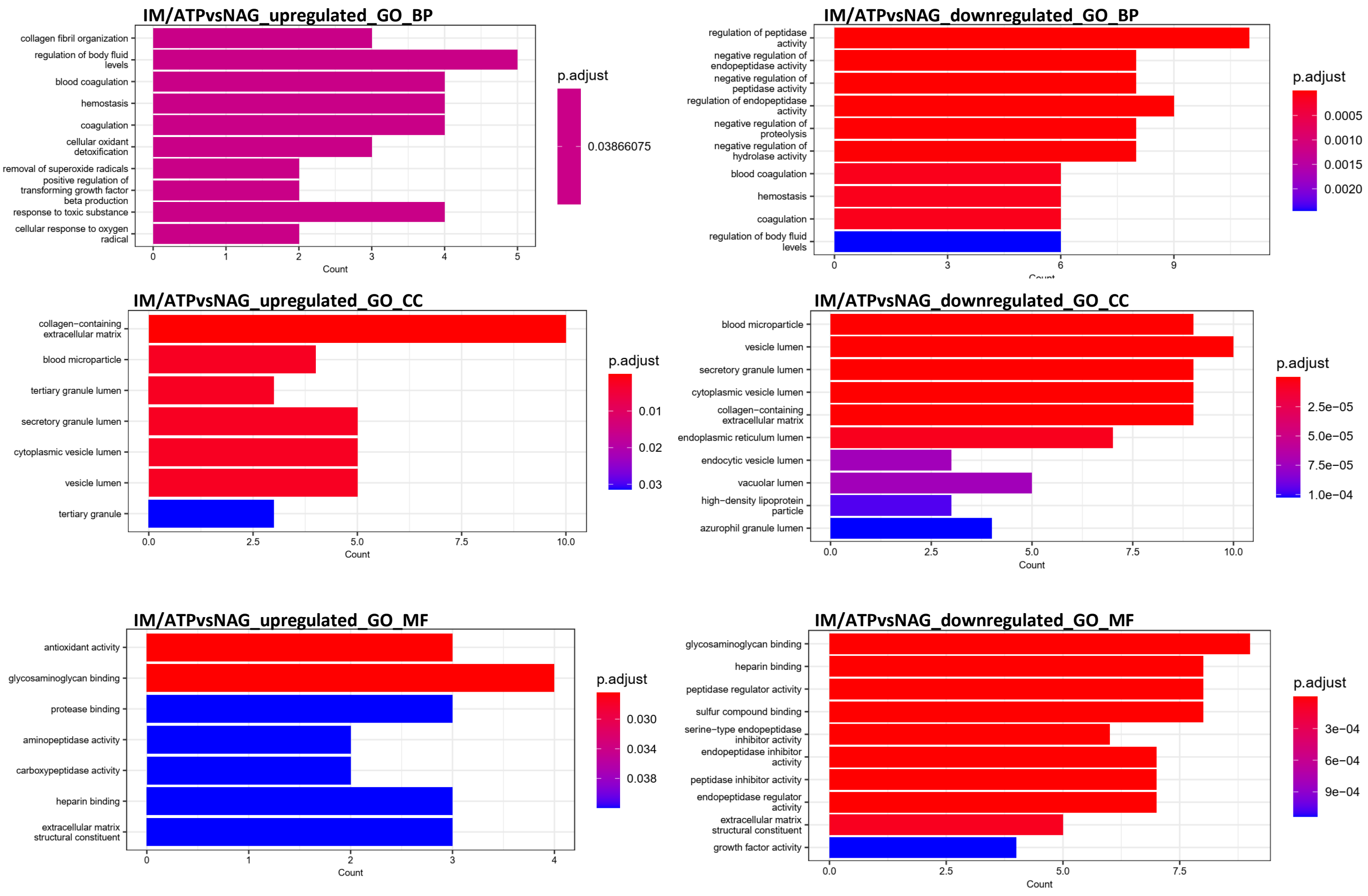

Figure S5. The traditional GO enrichment results of the IM/ATP vs NAG group.

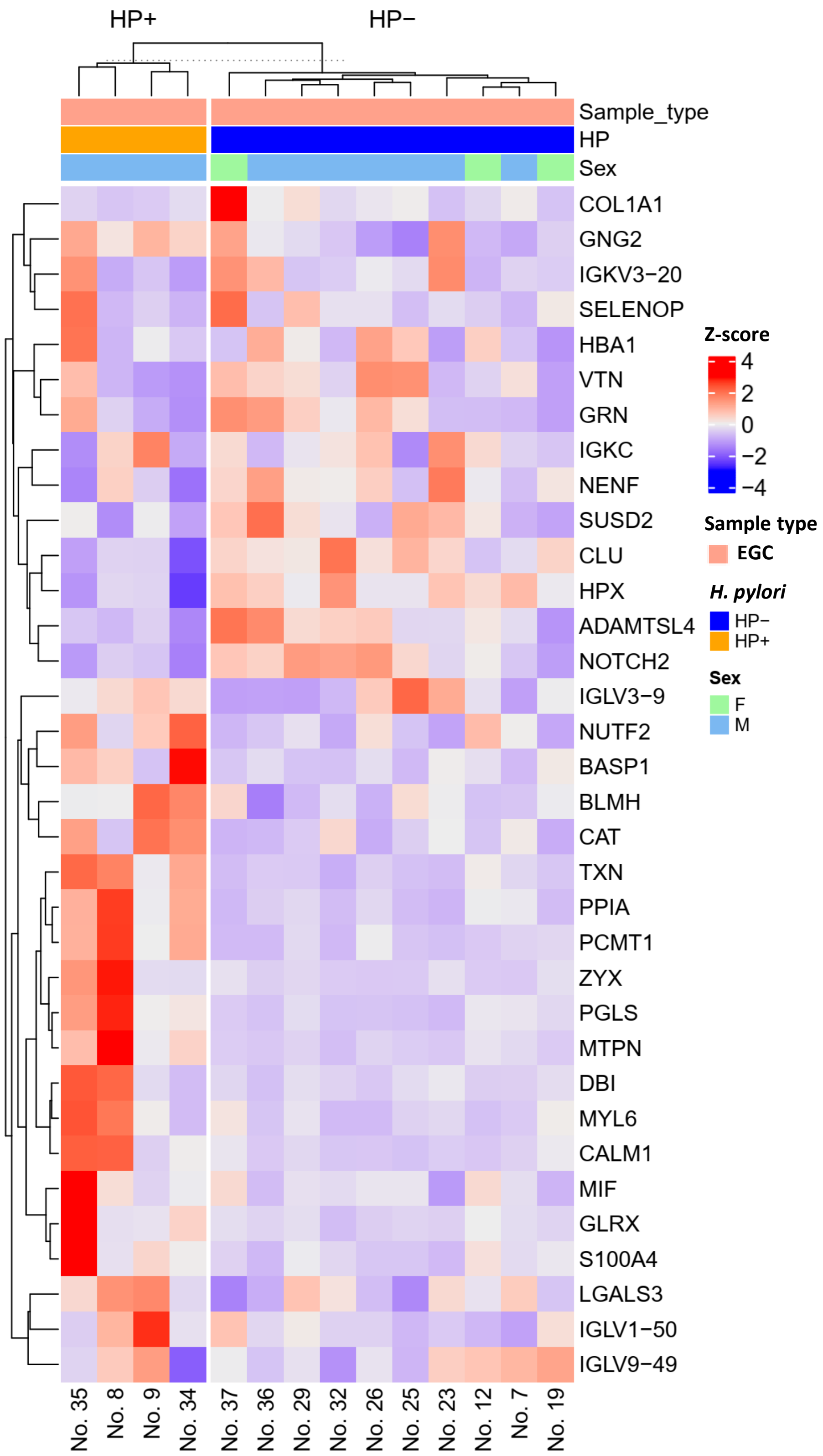

Figure S6. Heatmap of differentially expressed proteins for the *H. pylori*-positive EGC vs *H. pylori*-negative EGC comparison group. HP is short for *H. pylori*.

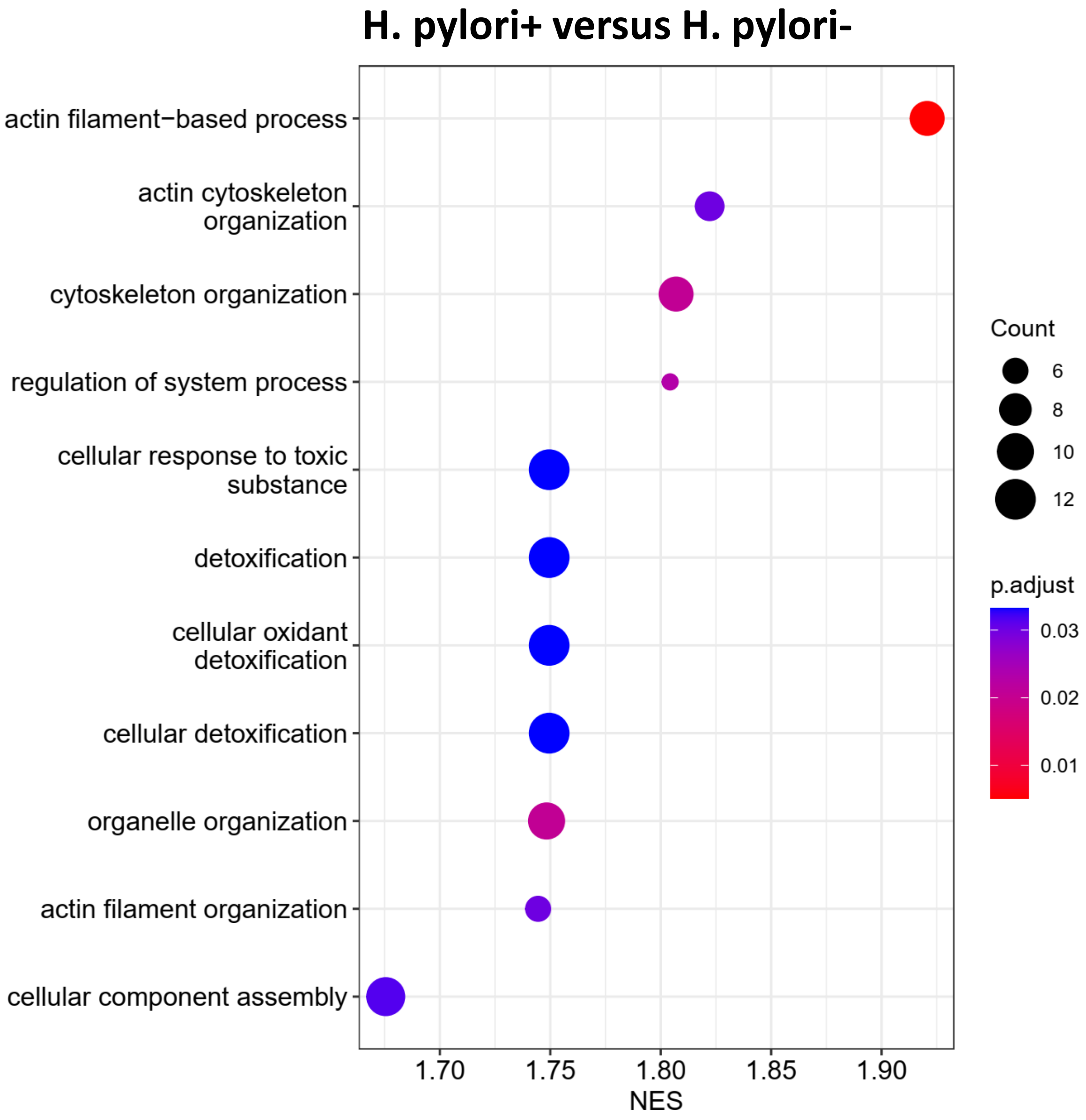

Figure S7. The enriched GO-BP gene sets by GSEA. The significantly enriched pathways (adjusted P value < 0.05) are listed.

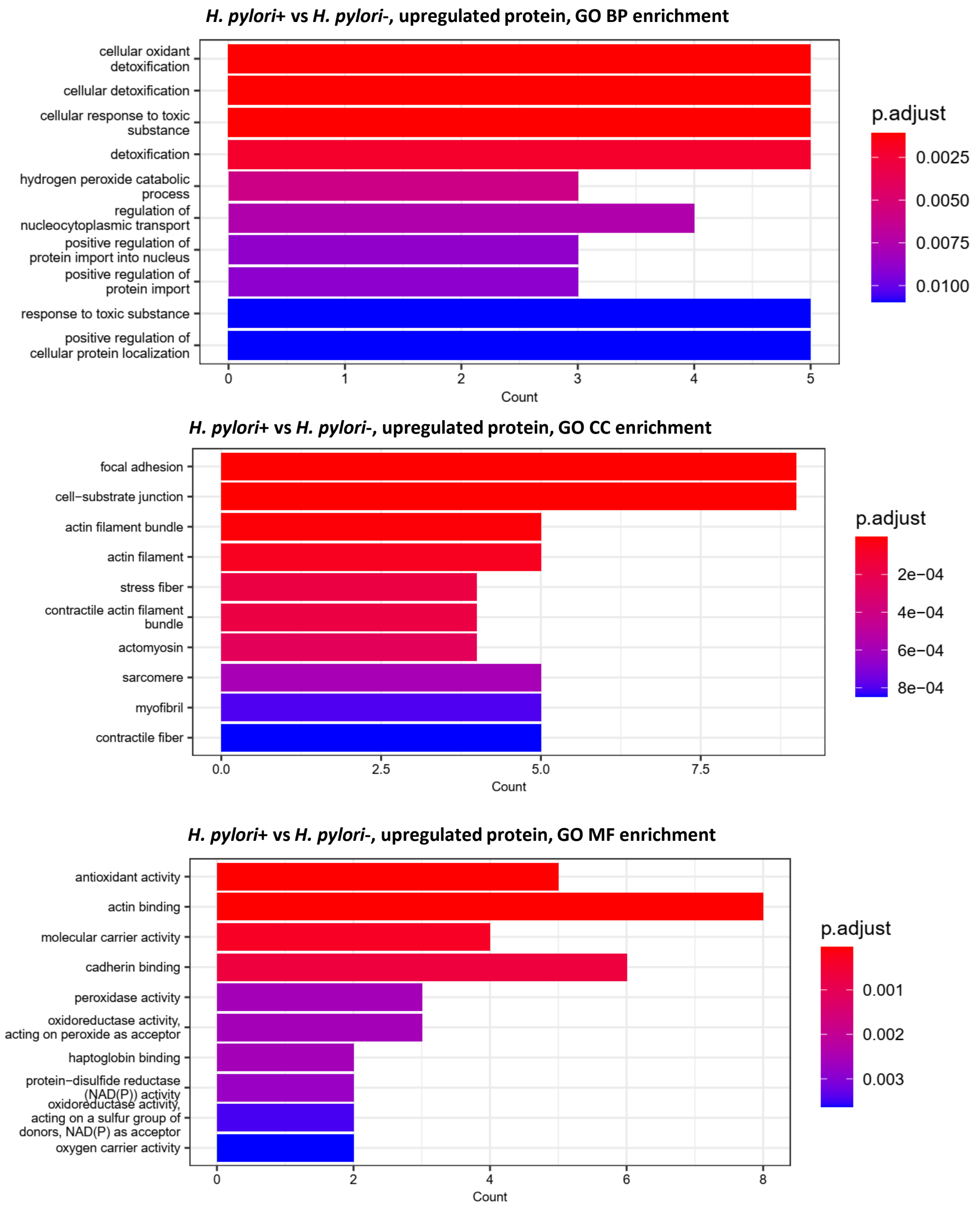

Figure S8.

The traditional GO enrichment results of the *H. pylori*-positive EGC vs *H. pylori*-negative EGC group.

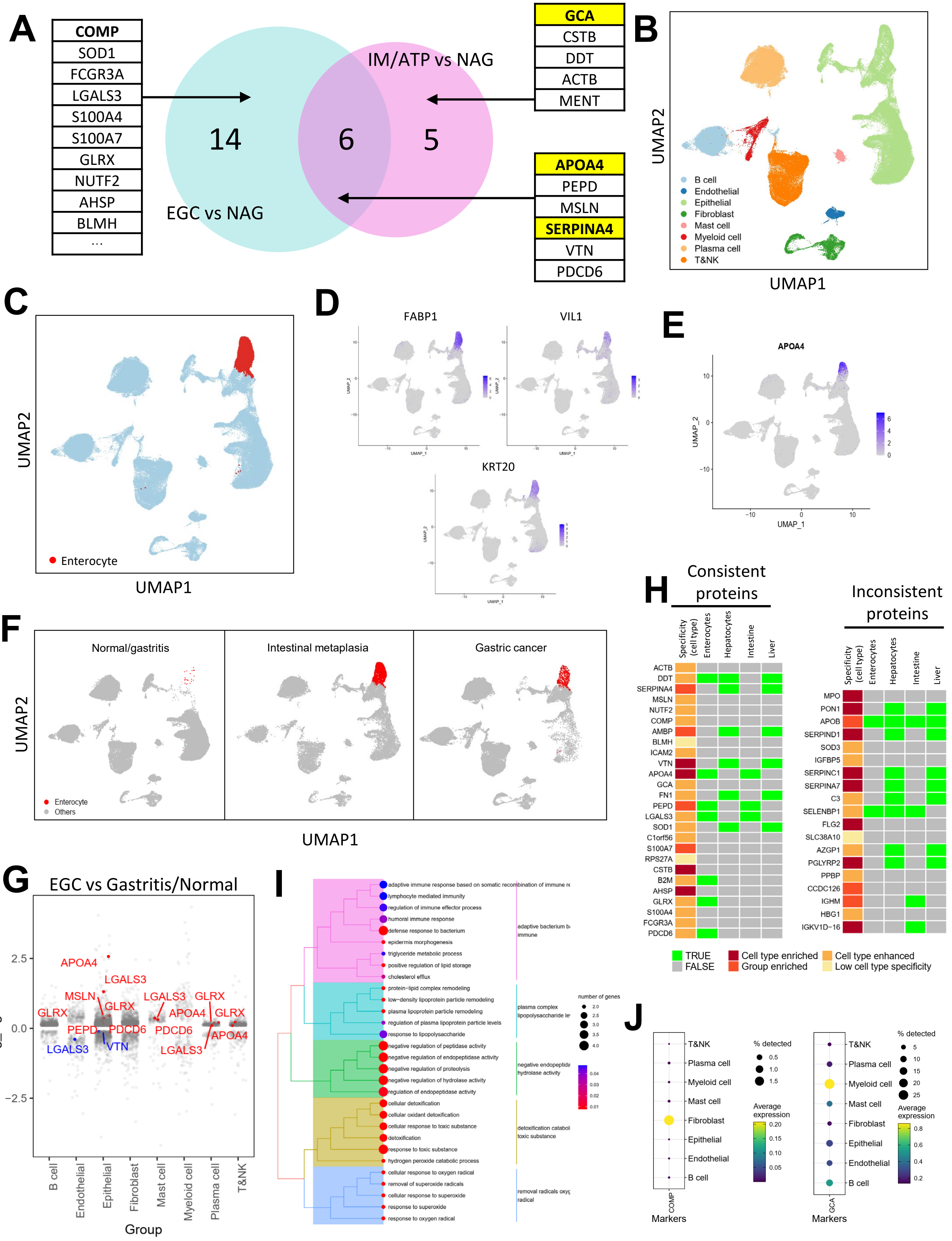

(legend on next page)

Figure S9.

Expression specificity of DEPs in single-cell RNA data and HPA database.

(A) Cell groups of the integrated data of GSE134520 and GSE150290.
(B) Enterocytes in the single-cell RNA data.
(C) Conserved marker genes of the enterocytes.
(D) APOA4 was high expressed in enterocytes.
(E) Enterocytes mainly exists in pre-GC and GC tissues, and hardly exist in nomal tissues.
(F) Consistent proteins of both the EGC vs NAG and IM/ATP vs NAG comparison groups.
(G) Fold changes of DEPs across cell types in GC vs gastritis/normal comparison group.
(H) Expression specificity of DEPs in Human Protein Atlas data;
(I) Enrichment analysis of the inconsistent proteins
(J) COMP is mainly expressed in fibroblasts, while GCA is mainly expressed in myeloid cells.

.
